# Migration of highly-skilled workers: personal perspectives

**DOI:** 10.11604/pamj.2022.41.292.34644

**Published:** 2022-04-12

**Authors:** Obinna Ositadimma Oleribe, Rodrigo Amo de la Fuente

**Affiliations:** 1 Best Health Consult Limited Liability Company (LLC), Klamath Falls, Oregon, United States of America,; 2 Royal Sussex County Hospital, Brighton, United Kingdom

**Keywords:** Economic migration, political migration, highly-skilled workers

## Abstract

Recently, one of the authors and his family migrated from Nigeria to take up employment in the United States of America and the other from Spain to the United Kingdom. Both journeys have been ones of mixed feelings, as we have both reaped rewards, but have experienced significant hardships. The migration of skilled workers has been ongoing for centuries and seems set to continue. Individuals who embark on such journeys become entangled with a lot of hopes and expectations, some of which may be unfounded. However, there are several benefits and drawbacks to migration from source nations into any of the advanced receiver countries. In this paper, we share our experience as highly-skilled workers and hope that this will help stimulate other individuals who have embarked on this journey or are yet to commence the process. We also hope that this paper will inform policy direction, employers´ attitudes, and community response to highly-skilled immigrant workers.

## Perspective

International migration is defined as the movement of people from one country to another to take up employment, establish residence, or seek refuge from persecution, either temporarily or permanently [1]. It is usually an exciting experience for an individual to migrate to any of the Organisation for Economic Cooperation and Development (OECD) mostly developed nations like the United States of America. The International Labour Organization (ILO) estimates the number of migrant workers at 105 million worldwide [2]. This is facilitated by the fact that most OECD developed countries expect growing shortages of highly-skilled labour in the coming decades, and have seen immigration as one way of addressing this problem, leading to the introduction of policies aimed at facilitating the recruitment of such workers in recent years [3]. A number of countries have loosened their requirement for migrant workers, making it easier for people to migrate to live and work in their nations, such as in China [4].

However, migration is not all from the global south, as some OECD developed countries such as Spain, Italy, Portugal and Greece overproduce highly-skilled graduates themselves and therefore these workers are added to the list of economic migrants to countries such as the United Kingdom in order to maintain skills and have the career to which they have been trained. Migrating to countries such as the USA or the United Kingdom has its potential benefits and hardships. Highly-skilled workers may migrate to enjoy a better lifestyle, secure income, education for their children and more opportunities in general [5]. Some migrate to study and work. Others migrate to stay permanently and then obtain a job once the necessary approvals are obtained. There is another group that get employed, for example, in a foreign-owned company and after a while, are transferred to the mother country to work. For this latter group, migration may be less difficult as these immigrants may have had prior exposure to work environments, customs, and traditions of the new system, and are thus more conversant with the expectations of the receiving country. Admittedly, there is a very small group of individuals who may have never worked with a receiving-country organization, but who may be privileged to get employed by such an organization and can work upon arrival in the USA or UK, for example. This group usually comprises highly-skilled individuals who are employed to bring added value to the USA or UK system upon arrival. They may be skilled in health management, healthcare, computer and ICT, engineering, and several other fields. This group of migrants either have a work permit or are helped by their host organizations to obtain work and resident permits.

All immigrants (low, middle, and highly skilled) are naturally affected by and may suffer different levels of cultural shock on arrival at their new employment site in the host country. To this group of workers, understanding the new work environment, cultural practices, and expectations, and the “wiring” of people´s minds in the new country would be most helpful - especially in the early stages of their cultural adaptation. Migrant workers are faced daily with a lot of challenges and abuse which affect their health, productivity, and longevity in the workplace. These challenges may range from language difficulty, cultural differences, values and beliefs, racism and lateral oppression. Although, many organizations claim to be equal opportunity systems in line with USA or European labor laws, in practice, this is not the case as there are several forms of inequity and unfairness to migrant workers. These may be system-engineered or policy-medicated. Until now, most OECD developed countries have largely ignored the challenges and abuse within the industry concerning migrant workers, but they may not be able to continue to do so for much longer [2].

Although immigrants may have different levels of skills, this article focuses mainly on highly-skilled legal immigrants who may be so classified because of having a tertiary degree or earning above a prescribed income limit. The recruitment and engagement of highly-skilled migrant workers is usually triggered either by demand or supply factors [3]. In demand-driven recruitment, immigrants are engaged by employer request, while in supply-driven recruitment, highly-skilled workers are invited to apply for a particular position, and the good ones are selected on the basis of certain characteristics, among them age, educational attainment, language proficiency, and occupation, for which points are assigned [3]. Candidates with qualifications and work experience obtained in an OECD-developed nation, like the USA, are preferred for recruitment for higher-paid positions. This may have informed the introduction, by many OECD developed nations, of policies and measures that allow international students to stay on after they complete their tertiary studies, provided they can find work of an appropriate level in their field of study [3]. When the need is critical, the employer may even facilitate work permits, cultural training, and language classes if native languages are used in the workplace.

The majority of these international migrant workers work under conditions that increase their risk of illness and injuries [6]. Every migrant or intending immigrant faces these challenges to different degrees. We are documenting issues from our own experience as highly-skilled migrants to highlight the need for acculturation in foreign lands.

### Advantages and drawbacks of high-skilled immigrant workers

**Qualifications and certifications issues:** to be qualified to be employed as a highly-skilled immigrant, one must either have a tertiary degree or a unique skill set that is needed to solve a particular problem, create a new product, or improve an existing system. This qualification and/or skill may be scarce in the recipient country. But, no matter the qualifications, as a migrant one may be viewed with suspicion. There is also a form of qualification racism as immigrants may suffer from a lack of skills recognition and employment commensurate with qualifications and experience [7]. These unsatisfactory formal and informal recognition processes result in subtle forms of racism. Therefore, even when not requested in the vacancy announcement, beyond diploma certificates, candidates may be asked to submit supporting documents, such as their transcripts and other evidence of scholarship. Submitting all these early in the engagement process may mitigate investigations that may be prolonged to confirm qualifications, improve bargaining powers, and ensure minimal discrimination and disruption of services. The more qualifications there are, the more important this is as a requirement for full absorption and engagement into the system at the appropriate level and pay scale. In the case of one of the authors (OO), proactively presenting transcripts at the point of employment would have saved his employers the headache of five weeks review and validation of his qualifications. In the case of the other author (RAF), political and healthcare-related inefficiencies combined to halt efficient processing of documentation, owing to Brexit and COVID-19 happening at the same time in the United Kingdom.

**Cultural difference issues:** highly-skilled individuals who migrate to various OECD developed countries (such as United Kingdom, United States of America, Australia, Germany, Sweden and France) from developing countries (such as India, Pakistan, Nigeria, Mexico and the Philippines) for work, experienced different levels of cultural shock - this is the mixture of anxiety and feelings of confusion, excitement, and insecurity to which each person feels differently [5]. These cultural shocks resulting from the unfamiliar environment with a completely different culture in a foreign country may be accompanied by familial, professional, and technical issues, along with the cultural shock. These have their positive and negative impacts, various forms of failure or struggle, and challenges to acclimatize to the appropriate host culture in the receiving organization, resulting in adjustment issues, owing to the difference in culture, lack of experience, and different lifestyles. These problems face immigrants working in a completely different set-up with new rules and regulations [5]. High perceived stress caused by inadequate living conditions, coupled with high cultural shock, high feelings of social isolation, and high discomfort from lack of understanding of the national language may all have negative effects on migrant workers' mental health [8]. Every highly-skilled immigrant should be fully aware that there will be major cultural differences, and this may affect their acceptance, acculturation, workflow, and outcome.

Language is another dimension of cultural shock that high-skilled immigrants face regularly. They may be poor or inadequate in the use of local dialect, requiring their attending language classes before officially resuming work. In the United States, although English is the official language, there are several tribal languages that an immigrant working with native Americans (for instance) will have to contend with. Furthermore, although all may speak the same language (English), certain words may have different meanings for different people. This may be more problematic for migrant workers from non-English speaking backgrounds (NESB) [9]. Language differences along with several other co-factors may result in several stress factors including difficulties with communication, unfamiliarity with the new environment and culture, work-related stress, practical stress, and social stress leading to decreased health, particularly with respect to psychological and psychosomatic distress [10]. In the experience of one of the authors (OO), requesting language and cultural awareness classes would have further empowered him with skills on various culturally sensitive issues. In the experience of the other author, RAF, being in limbo while awaiting UK document accreditation during the COVID-19 pandemic allowed time for language schooling, but in order to pay for this, he had to work in a coffee shop and then as a cleaner in the local hospital, despite his highly-skilled status.

**Ethical issues:** these vary remarkably across nations of the world, as what is ethically correct and or accepted in one region, state or nation may be unacceptable in others. Most high-skilled immigrants have four main ethical issues in the United States or Northern Europe - privacy, accessibility, property, and accuracy [11]. For instance, how much personal information should a migrant reveal to employers, or those who ask, and under what condition (privacy)?; what information and to what level does an organization have the right to seek and obtain, and under what conditions and safeguards (accessibility); who owns this information and the channels of their communication (property)?; and who is responsible for the fidelity, accuracy, and authenticity of this information, and who is to be held accountable for errors in the accuracy of the information, and how will the injured be made whole (accuracy) [12]?

**Cost of living issues:** highly-skilled immigrant workers spend more in their new location to settle down, equip their homes, buy food, and move around. It is not uncommon to see them live first in hotels, then in a temporary, but expensive accommodation, before qualifying to rent. Buying houses are also impossible initially as they do not have 12 to 24 months´ rent history, nor a good credit history if this is their first entry into the United States or the United Kingdom. New immigrants may have to hire or rent vehicles for transportation, buy furniture, fitting and equipment; and for those living in small cities, import what they need from distant, larger cities. To obtain their full documentation, driver´s license, state identity cards, social security number, and work permits, a lot of resources are needed and are spent. So, even when the employing agency decides to support these processes with relocation allowances, this is usually never enough to cover the expenses incurred by highly-skilled immigrant workers in traveling and settling down. One of the authors (RAF) had to work in the unskilled job sector in order to have an income while awaiting validation of degrees and training documents. This experience is not unique and highly-skilled migrants should be prepared to do similar to support themselves because with the COVID-19 pandemic efficiency of government agencies has been slowed to a halt in many countries.

**Capacity and documentations issues:** highly-skilled immigrants are expected to, not only have the required qualifications for the job but to really know their work and assignments. They should both “talk the talk” and “walk the talk”. They are not expected to think they know their work, but to really know the work as people will expect the very best from them. The employing organization will also expect the new migrant to go “above and beyond” in their task, work beyond the normal work hours to deliver outcomes that are second to none, and have extraordinary results to justify employment. However, because highly-skilled migrants often have a different experience from that of native skilled workers, their perspectives on problem-solving and access to non-overlapping knowledge networks will differ from natives [13]. This difference in perspective makes migrant hires a particularly valuable resource in the context of organization-level innovation if properly exploited to the advantage of the system. They can contribute to organizational innovation performance that new highly-skilled local or migrant hires who do not add cultural diversity may not [13]. To get the best from employing highly-skilled migrants in terms of innovation-related benefits, organizations should increase their integration capacity.

Highly-skilled immigrants will be compared naturally with natives who had previously occupied the same office and positions. As staff may wonder why the migrants were employed in the first place, seeking reasons to work for or against them, it is the capacity and documentation skills of the migrant workers that will distinguish them and solidify their position. It is also important to note that proponents of the human capital theory suggest that skill plays a key role in employment prospects for international migrants because of the more skilled the worker, the greater his/her productivity [14]. Moreover, productive workers enjoy better jobs and mobility in the labor market. Critics argue that a policy emphasis on migrants' skill levels tends to simplify the employment and broader socio-cultural challenges migrants face.

One of the unique capacities expected of a highly-skilled migrant is the capacity for elaborate documentation of activities, communications, decisions, and action. To mitigate litigation and backlash, a highly-skilled worker should ensure that all the Ts are crossed, and Is are dotted. For instance, to engage new staff, a migrant worker must document every single step taken from vacancy announcement to selection and onboarding. If this is not properly done, the new immigrant may lose in a court of law if they believe that they were not taken because of irregularities, bias, or policy violations. To introduce new concepts and practices, a skilled immigrant must ensure that every step is equally documented, and that required approvals are obtained from superior powers before next steps are taken, even when there are policy provisions. This becomes more important when such policy provisions have never been implemented. Deliberately proactively seeking clear approval will prevent these conflicts.

**Character and core values issues:** a lot is expected of a highly-skilled immigrant worker - far and above the native and locally trained/employed workers. Character and core values are key to a sustainable placement. Both employer and employees are looking for a slight slip especially if the migrant is from a country noted for corruption and other malpractices. People will check the migrant out, weigh them in the balance, and make judgments concerning them. Their character in terms of time management, meeting management, professionalism, personality type, human relationships, and several other character aspects will determine how the migrant fits into the system, and how long they will keep their current position. It is advisable that a single-line narrative be avoided in judging and assessing a highly-skilled migrant worker.

**Racism, lateral oppression, and gender issues:** highly-skilled immigrants working in their native countries enjoy protection against all forms of racism and lateral oppression. However, this is completely lost as one migrates into a receiver nation, such as the USA. This is worst if one is to work in a state or location that has a uniformity of a particular ethnic group, such as Caucasian or native tribes; or states with significant white supremacy, as may be seen in certain parts of the USA.

**Supervision issues:** highly-skilled immigrant workers are also faced with having supervisors with different orientations, values, and work ethics. This is worst when the supervisors are less qualified, may not have tertiary education, and may even suffer from low self-esteem or ego problems, historical trauma, or are currently in need of psychological or mental health support. It is not also uncommon to have these highly-skilled immigrants supervised by multiple individuals with a different understanding of the primary task, assignment, and goals. This may result in abusive supervisory practices in the workplace that have been shown to have important direct consequences in work and work relationships, and also indirect consequences on workers´ well-being and relationships outside work [15]. In some instances, because migrant workers´ status in the host country of work is dependent on maintaining the work contract, they develop a coping mechanism to the negative indirect consequences of abusive supervision which may affect different aspects of their well-being adversely, resulting in work-related stress and devastating work-life balance [15]. These supervisory issues are worse for highly-skilled migrant workers who were self-directed, self-led, and self-supervised; or who had supervisors with the same training, the same goals and the same core values in their native countries. There is the need for migrant workers to think about these physical and psychosocial dynamics, and plan for them as they prepare to emigrate.

**Ways forward:** migrant highly-skilled workers must develop a new order of resilience and stable mental health to ensure better quality of life and faster adaptation in their new countries [8]. Higher work burden and lack of rest, comparatively lower wages, poor living environment, low economic status and deficiency of living necessities, hard physical labor, and conflicts with native workers have negative effects on physical and mental health of immigrant workers [8]. To improve the mental health of migrant workers, social support including financial support and educational programs that foster resilience is needed, and should be provided to all highly-skilled immigrant workers.

To achieve this, it is important that countries improve their ability to measure migration patterns by country of origin to determine the training needs and other assistance required and engage employers appropriately [2]. Due to high acculturative demands and increased vulnerability, highly-skilled migrant workers should be recognized as a specific target group for health promotion and health services [10]. There is also the urgent need for fresh, empathic and bold approaches and initiatives to promote the health and welfare of highly-skilled immigrant communities, and their families in the United States of America, in particular, and the global space at large [12].

Furthermore, there is the need to see to the full implementation of the codes of practice and bilateral memoranda of understanding established in 1999 which strives to protect the rights of migrant workers, ensure the provision of adequate workplace support for migrant workers and seek to ensure that migration flows do not disrupt health services in source countries [1]. Although there is no agreed definition of ethical international recruitment, and no consensus on the significance and location of harmful recruitment practices, substantial migration and recruitment has occurred outside the scope of this code of practice, and codes have diverted skilled health workers beyond regulation. While we suggest that recipient countries work at ensuring the production of adequate numbers of highly-skilled workers, source countries should focus on working to improve employment conditions for highly-skilled workers. The world must also work towards ensuring the health and wellbeing of those who have migrated or are in the process of migrating to provide the much-needed services in the recipient nations.
